# Factors Predicting Willingness to Share COVID-19 Misinformation

**DOI:** 10.3389/fpsyg.2020.566108

**Published:** 2020-09-24

**Authors:** Emilio J. C. Lobato, Maia Powell, Lace M. K. Padilla, Colin Holbrook

**Affiliations:** ^1^Department of Cognitive and Information Sciences, University of California – Merced, Merced, CA, United States; ^2^Applied Mathematics Department, University of California – Merced, Merced, CA, United States

**Keywords:** conspiracy theories, COVID-19, misinformation, social media, political orientation

## Abstract

We conducted a preregistered exploratory survey to assess whether patterns of individual differences in political orientation, social dominance orientation (SDO), traditionalism, conspiracy ideation, or attitudes about science predict willingness to share different kinds of misinformation regarding the COVID-19 pandemic online. Analyses revealed two orthogonal models of individual differences predicting the willingness to share misinformation over social media platforms. Both models suggest a sizable role of different aspects of political belief, particularly SDO, in predicting tendencies to share different kinds of misinformation, predominantly conspiracy theories. Although exploratory, results from this study can contribute to the formulation of a socio-cognitive profile of individuals who act as vectors for the spread of scientific misinformation online, and can be useful for computationally modeling misinformation diffusion.

## Introduction

Currently, the world is experiencing a global pandemic of SARS-CoV-2, the virus causing the COVID-19 disease (World Health Organization, 2020). Scientific and medical information concerning the virus is being discovered and relayed quickly in efforts to inform the general public and policymakers about how best to respond. The demand for information related to COVID-19 is high, creating a prime environment for misinformation to spread.

The information environment surrounding the pandemic affords an opportunity to study the spread of scientific misinformation on social media platforms. We explored whether different patterns of individual differences predict the inclination to share different kinds of misinformation about a salient socio-cultural scientific topic. For the purposes of the present research, we limited our focus to individual differences in propensity toward conspiracy ideation, attitudes toward science, and facets of political ideology. Each of these individual differences has been previously found to relate either to the endorsement of misinformation or to how people respond to health threats from pathogens, as will be briefly described below.

## Misinformation Diffusion Online

Research on the diffusion of information online consistently finds that misinformation diffuses faster and reaches broader audiences than correct information (del Vicario et al., 2016; Vosoughi et al., 2018). Exploring information sharing over social media platforms can facilitate the scientific understanding of the spread of misinformation. Here, we focus on factors associated with willingness to disseminate misinformation online. It is important to note that spreading misinformation does not need to be indicative of a deliberate attempt to deceive nor does spreading misinformation necessarily stem from a person being gullible. Sharing misinformation online can occur under a variety of other circumstances, such as when people post a link to an article to try and generate discussion among their social network or to draw attention to a misinformed claim as being misinformed. The current work does not focus on the specific motivations people may have for sharing misinformation, but rather the overall willingness to share claims regarding the current COVID-19 pandemic that happens to be untrue or unverifiable over social media.

Prior research investigating who shares misinformation on social media suggests that older individuals and people who are more politically conservative tend to share more political misinformation online relative to younger individuals, liberals, or moderates (Guess et al., 2019). Additionally, individuals who tend to gravitate toward conspiracy narratives on social media platforms are more likely to positively engage with – in the form of “likes,” sharing, and commenting – misinformation claims than are individuals who gravitate toward scientific narratives (Bessi et al., 2015). Much of the recent research examining the spread of specific information and misinformation over social media has focused on sharing political information, mostly surrounding elections (e.g., Buchanan and Benson, 2019; Guess et al., 2019; Mosleh et al., 2020). However, relatively scant research has examined how these platforms are used for sharing and spreading information on specific scientific topics. By focusing on COVID-19 misinformation, the present research contributes to understanding the spread of misinformation on a specific scientific topic, albeit a scientific topic that has come to intersect with politics.

## Individual Differences Pertaining to Misinformation

Conspiracy theorists typically posit explanations for large-scale events that contradict official or expert explanations (Goertzel, 1994). They tend to be distrustful of recognized legal or scientific cultural authorities. This distrust of authority is so pervasive in conspiracy ideation that people inclined to believe conspiracies will accept mutually exclusive conspiracy theories more than the official account of a major socio-cultural event (Wood et al., 2012). On social media, groups focused on disseminating conspiracy-related content – frequently framed as trying to inform people of news not covered by the mainstream news – tend to be more active than groups focused on disseminating scientifically informed content (Bessi et al., 2015). Accordingly, we are investigating the influence of individual differences in conspiracy ideation on willingness to share misinformation.

Researchers have found that belief in conspiracies correlates with the rejection of science and endorsement of pseudoscience (Lewandowsky et al., 2013a,b; Lobato et al., 2014; van der Linden, 2015; Lobato and Zimmerman, 2019) and to a general attitude toward science as lacking credibility (Hartman et al., 2017). Misinformation pertaining to how COVID-19 spreads, how susceptible different groups are, and what kinds of treatment or prevention methods are effective can emerge and spread from individuals who are antagonistic toward rigorous scientific investigation or those with financial or other incentives at odds with scientific rigor. Relatedly, information and misinformation about COVID-19 that is being disseminated frequently takes the form of empirical claims or interpretations of the results of preliminary empirical investigations (e.g., the headline “Some Blood Types May Be Slightly More Susceptible to COVID-19, Paper Suggests” from Bowler, 2020). Therefore, understanding who is likely to spread misinformation about a scientific topic requires assessing attitudes about science in general.

Because the COVID-19 pandemic represents a pathogen threat, research on individual difference factors related to pathogen threat responses is relevant. Convergent studies provide evidence that political conservatives are relatively more disgust-prone than are liberals, an affective response theorized to functionally relate to pathogen avoidance (Inbar et al., 2012; Terrizzi et al., 2013). Tybur et al. (2016) conducted a large multinational study to compare two theoretical accounts of the apparent positive correlation between pathogen sensitivity and political conservatism. According to one account of this relationship, which Tybur and colleagues call a “traditional norms” account, some cultural traditions and behavioral norms (particularly surrounding food preparation) arise because they help neutralize threats posed by pathogens. Under this model, the link between pathogen sensitivity and political conservatism is driven largely by adherence to the traditional moral values and lifestyles of the in-group. A distinct intergroup account of the relationship between political views and pathogen stress response, which Tybur and colleagues call an “out-group-avoidance” account, posits that over time individuals develop resistance to local pathogens but remain vulnerable to pathogens borne by out-group members. Under this account, the relationship between pathogen sensitivity and political views is driven primarily by ideologies favoring hierarchical social stratification, termed social dominance orientation (SDO; Pratto et al., 2013), that place out-groups in subordinate positions. Tybur et al. (2016) tested both accounts in cross-cultural research spanning 30 nations, finding support for the traditional norms account over the out-group-avoidance account. Although inclinations toward social dominance and adherence to traditionalism are both associated with political conservatism, pathogen-avoidance responses appear to be driven more by traditionalism than social dominance. Here, we include both measures of SDO and traditionalism to explore their relative contributions to the spread of health-related misinformation in the midst of a global pandemic.

In sum, prior research provides evidence that interrelated dispositions may be related to conspiracy ideation, negative attitudes toward science, and political ideology. Further, these factors may also predict willingness to share misinformation. The goal of the present exploratory research is to begin characterizing the socio-cognitive profile of individuals likely to spread misinformation online. To achieve this goal, we questioned individuals about their willingness to share COVID-19 misinformation over social media platforms and took measures of their inclination to conspiracy ideation, their attitudes toward science, and their political ideology along several dimensions. Materials, data, and study preregistration documents are available on the Open Science Framework: https://osf.io/ytsr8/.

## Materials and Methods

### Participants

We recruited 404 participants *via* Amazon’s Mechanical Turk, comparable to other research on credulity about hazard claims (e.g., Samore et al., 2018). We removed data on the basis of preregistered criteria: incomplete responses to the dependent measure or individual difference measures, completing the study in less than 2 min, and failure to respond or nonsensical response to an open-ended question asking them to describe the study. The final sample, after exclusions, was 296 participants (*M_age_* = 36.23, *SD_age_* = 10.96; 178 men, 117 women, 1 other). Participants were paid $0.75USD for participation.

### Materials

We used fact-checking sites, such as Snopes.com and FactCheck.org, to create an *ad hoc* measure of peoples’ willingness to share misinformation about COVID-19 over social media. Eighteen actual claims, either verified to be untrue or unverifiable, that have been made regarding COVID-19 were presented to participants. For each claim, participants used a slider to indicate how likely they would be to share that claim over their social media accounts. The slider bar ranged from scores of 0 to 100, with anchors of “Definitely not share,” “Less likely to share,” “More likely to share,” and “Definitely share” located at the 0, 33, 66, and 100 marks, respectively. We calculated mean scores for participants’ willingness to share misinformed claims about COVID-19. The items selected for this scale were *a priori* categorized as claims regarding: (a) severity and spread of COVID-19 (*α* = 0.91), (b) treatment and prevention of COVID-19 (*α* = 0.92), (c) COVID-19 conspiracy theories (*α* = 0.89), and (d) miscellaneous incorrect or unverifiable claims (*α* = 0.78). Table 1 details the sets of claims and categorization scheme. The categorization scheme utilized in the current work was based on the categorization structure of claims from the originating fact-checking sites and was conducted by two authors. For example, Snopes.com created multiple webpages for fact-check coronavirus claims (available here: https://www.snopes.com/collections/new-coronavirus-collection/). The categorization scheme in this study was inspired by categorizations used on Snopes.com: “Origins and Spread,” “Treatment and Prevention,” and “Conspiracy Theories.” We build on this by including a “Miscellaneous” category which includes claims from diverse categories on the Snopes collection webpage, such as “Media and Entertainment” or “Prophecies and Predictions.”

**Table 1:** COVID-19 misinformation claims used in the study.

Severity/Spread	1. Health experts predicted the new coronavirus could kill 65 million people.
	2. Chinese doctors confirmed that African people are “genetically resistant” to new coronavirus.
	3. Warmer weather will inhibit the spread of the new coronavirus.
	4. The novel coronavirus COVID-19 is more deadly than any known pathogen.
	5. Only the elderly and people with preexisting medical conditions can catch the coronavirus.
	6. People with Type-A blood are more susceptible to COVID-19.
Treatment/Prevention	7. Taking a few sips of water every 15 min will prevent the new coronavirus from entering your windpipe and lungs.
	8. If you can hold your breath without coughing, discomfort, stiffness, or tightness, your lungs do not suffer from fibrosis and therefore you have no COVID-19 infection.
	9. Mass vaccination for COVID-19 in the African country of Senegal was started April 8th and the first seven children who received it died on the spot.
	10. Lemon Juice Tea has been shown to cure COVID-19.
Conspiracies	11. Democrats in New York stashed ventilators in a warehouse in an effort to make the COVID-19 pandemic worse.
	12. The COVID-19 virus is a chimera. It includes SARS, an already weaponized coronavirus, along with HIV genetic material and possibly flu virus.
	13. Donald Trump owns stock in a company the CDC uses for COVID-19 tests.
	14. 5G cellular service technology is linked to the cause of the coronavirus.
	15. COVID-19 was created in a virology lab as a potential bioweapon, but accidentally got released before it had been fully studied by its creators.
Miscellaneous	16. Sales of Corona beer dropped sharply in early 2020 because consumers mistakenly associated the brand name with the new coronavirus.
	17. Idris Elba and other celebs have been paid to say they have coronavirus.
	18. Nostradamus predicted the COVID-19 pandemic.

### Individual Difference Measures

We measured participants’ disposition toward conspiracy ideation with the Conspiracy Mentality Questionnaire (*α* = 0.83; Bruder et al., 2013). Participants rated their level of certainty about various statements on an 11-point Likert scale (0% – *Certainly Not* to 100% – *Certain*). This five-item measure includes statements such as “I think there are secret organizations that greatly influence political decisions.”

We measured participants’ general attitudes toward science with the Credibility of Science Scale (CoSS; *α* = 0.94; Hartman et al., 2017). This six-item measure asks participants to respond on a 7-point Likert Scale (1 = *Disagree Very Strongly*; 7 = *Agree Very Strongly*) to statements such as “People trust scientists a lot more than they should.” The CoSS is scored such that higher scores represent less favorable views of science as credible.

We used a modified version of the Political Issues Index (*α* = 0.76; Dodd et al., 2012; Holbrook et al., 2018) as a proxy for where participants generally fall on the liberal-to-conservative political spectrum. This 20-item measure lists socio-political issues (e.g., “Same-sex marriage,” “Reduce business regulations,” and “Right to abortion”), and participants indicate whether they *Agree*, *Disagree*, or are *Uncertain* about the issue. The Political Issues Index is scored from −1 to 1, reverse-scoring agreement with the traditionally liberal items, such that lower values represent greater alignment with traditionally liberal policy positions, and higher values represent greater alignment with traditionally conservative policy positions (“Uncertain” responses are scored as zero).

We used the SDO short form (*α* = 0.74; Pratto et al., 2013) to measure approval of social hierarchies. Participants respond to this four-item measure by using a 7-point Likert scale (1 = *Extremely Oppose*; 7 = *Extremely Favor*) to indicate how much they reject or support statements concerning social hierarchies and egalitarianism. An example item is “Superior groups should dominate inferior groups.”

We used the six-item Traditionalism subscale from the Authoritarian-Conservatism-Traditionalism scale (*α* = 0.83; Duckitt et al., 2010) to measure participants’ valuation of traditional moral systems and lifestyles and resistance to modern challenges to such traditional values and lifestyles. Participants responded on a 7-point Likert scale (1 = *Strongly Disagree*; 7 = *Strongly Agree*) to statements such as “This country will flourish if young people stop experimenting with drugs, alcohol, and sex, and pay more attention to family values.”

### Procedure

After providing informed consent, participants were presented with the following instructions:

We are interested in examining what types of things people share over social media. Sometimes people share information because they think it is true and want others to know it. Sometimes people share information even if they think it is false because they would like to warn other people to not believe it if they hear it from somewhere else. Sometimes people share information that they are not sure about as a way to see what their friends and family think. And sometimes people share information for other reasons entirely.
In this task, you will be presented with a series of claims regarding the current COVID-19 (aka SARS-CoV-2) pandemic that have been made and shared over both traditional media outlets, such as TV news programs or newspapers, and over social media outlets, such as Facebook or Twitter. You may have even encountered some of these already.
For each claim, use the slider bar provided to rate how likely you think you would be to share this over your own social media accounts.

After reading the instructions, participants completed the task. The 18 claims we used as stimuli were presented in a randomized order. Participants were informed that these were real claims that have been made on both traditional news media outlets and on social media platforms. Following this task, participants filled out the individual difference measures in randomized order. Finally, participants filled out a demographics form. Participants were debriefed as to the nature of the study and informed that the claims they read regarding COVID-19 were not true. In the debriefing, we provided links to fact-checking and health agency websites for participants, to help provide participants with resources to keep up to date with COVID-19 information and misinformation.

## Results

Table 2 presents the descriptive statistics for scores on the individual difference measures and for mean participant ratings of their likelihood to share the examined types of COVID-19 misinformation. On average, our sample was not inclined toward liberalism or conservatism, as measured by the modified Political Issues Index. Our sample was mildly inclined toward conspiracy ideation. Additionally, the sample was mildly above the midpoint for the CoSS, indicating a slight inclination toward rejecting science as credible. Our sample also averaged slightly below the midpoint on the SDO scale, while averaging around the midpoint on the Traditionalism scale. Regarding willingness to share COVID-19 misinformation claims over social media, our sample averaged below the midpoint, suggesting an overall low willingness to share the COVID-19 claims we tested. All measures correlated significantly with each other at the *p* < 0.001 level; Table 3 shows the correlation matrix. Diagnostics for the inferential analyses reported below revealed no outliers that exerted sufficient influence on the models to warrant removal and that all assumptions necessary for linear analysis were met.

**Table 2:** Descriptive statistics.

	*M*	*SD*	Range	Skew	Kurtosis
Conspiracy Mentality Questionnaire	7.73	1.80	1–11	−0.91	−0.23
Credibility of Science Scale	4.25	1.70	1–7	−0.48	−0.94
Political Issues Index	−0.05	7.41	−20 – 20	−0.48	−0.23
Social Dominance Orientation	2.95	1.39	1–7	−0.08	−1.21
Traditionalism	3.86	1.39	1–7	−0.12	−0.27
COVID-19 claims total	41.67	27.31	1–100	0.19	−1.09
Severity/Spread	44.81	27.97	1–100	0.06	−1.08
Treatment/Prevention	38.48	31.53	1–100	0.24	−1.29
Conspiracies	40.39	28.47	1–100	0.17	−1.10
Miscellaneous	41.77	27.20	1–100	0.21	−0.93

*N* = 296.

**Table 3:** Pearson product moment correlations.

	1	2	3	4	5	6	7	8	9	10
1. CMQ		0.57	0.34	0.27	0.33	0.47	0.45	0.43	0.49	0.40
2. CoSS			0.58	0.54	0.59	0.62	0.57	0.58	0.65	0.54
3. PII				0.32	0.77	0.28	0.28	0.28	0.30	0.17
4. SDO					0.20	0.44	0.35	0.43	0.48	0.43
5. Traditionalism						0.35	0.36	0.35	0.34	0.21
6. COVID claims							0.96	0.96	0.96	0.89
7. Severity/Spread								0.89	0.88	0.81
8. Treatment/Prevention									0.90	0.81
9. Conspiracies										0.84
10. Miscellaneous										

*N* = 296. All correlations significant at the *p* < 0.001 level. CMQ, Conspiracy Mentality Questionnaire; CoSS, Credibility of Science Scale (higher scores indicating greater skepticism of science); PII, Political Issues Index (higher scores indicating greater conservatism); SDO, Social Dominance Orientation short form.

We assessed the relationship between the individual difference measures and self-reported willingness to share different kinds of COVID-19 misinformation over social media using a canonical correlation analysis. A canonical correlation analysis allows analysis of the relationship between sets of predictor and outcome variables by creating synthetic variates representing linear combinations of the predictor variables and linear combinations of the outcome variables. For each synthetic variate, the strength of the contribution to the synthetic variate for each variable produces a function coefficient. Additionally, the analysis produces a bivariate correlation between each predictor and criterion variable and the respective synthetic variate, known as the structure coefficient. This analysis strategy is designed to generate the highest correlation between the two variable sets (Tabachnick and Fidell, 2007). In canonical correlation analysis, multiple orthogonal models are created, equal to the number of variables in the smaller set. The first model is created to maximally explain the variance between the two sets of predictors, and subsequent models are created to maximally explain the remaining variance not explained by prior models. Each model represents one unique linear combination of outcome variables regressed onto one unique linear combination of predictor variables. We chose this multivariate analysis strategy because of the exploratory nature of the research, as it is an approach that can reveal at once multiple potential ways in which sets of variables relate to each other, rather than running a series of univariate multiple regression analyses. Canonical analysis is useful for exploratory research where there are distinct sets of variables of interest, such as a set of potential independent variables and a set of potential dependent variables.

The full model across functions was significant, creating four functions with squared canonical correlations (canonical *r*^2^) of 0.48 for the first function, 0.10 for the second function, 0.02 for the third function, and 0.01 for the fourth function. However, only the first function (Wilk’s *λ* = 0.45, *F*(20, 952.8) = 12.84, *p* < 0.001) and the second function (Wilk’s *λ* = 0.88, *F*(12, 762.3) = 3.16, *p* < 0.001) were significant, and combined explained 58% of the total variance. Sensitivity analysis conducted using G*Power (Faul et al., 2009) with power set to 0.90 and *α* set to 0.05 revealed our analysis was powered sufficiently to detect effect sizes as small as *f*^2^ = 0.056, corresponding roughly to *r*^2^ = 0.053.

**Figure 1:**
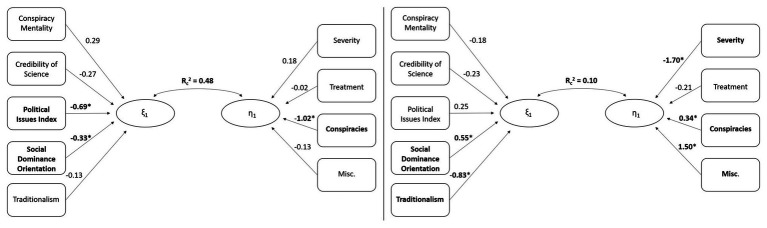
Diagram of the two significant canonical models. Substantial contributors to the synthetic predictor variate (*ξ*) and criterion variate (*η*) are bolded and noted with *. The squared canonical correlations (*R*^2^_c_) are significant at the *p* < 0.001 level. **Left panel:** More alignment with liberal policy positions and a low social dominance orientation (SDO) predict a low willingness to share conspiracy theories about COVID-19 on social media. **Right panel:** A high SDO and a low endorsement of traditionalism predict a low willingness to share misinformation on social media related to the severity and spread of COVID-19, but a high willingness to share conspiracies about COVID-19 and miscellaneous cultural misinformation about COVID-19.

For the first function (see Table 4), the synthetic predictor variate was primarily composed of participant scores on the Political Issues Index and the measure of SDO, possessing standardized function coefficients greater than |0.33|. The first synthetic criterion variable was primarily composed of participant’s intention to spread conspiracy-related misinformation, with a standardized function coefficient of −1.02. Together, the first model reveals that participants who are primarily more liberal (in terms of the issues index) and less oriented toward social dominance were less inclined to share COVID-19 claims that were conspiratorial in nature (see Figure 1). Additionally, the standardized structure coefficients revealed that all individual differences significantly correlated with the synthetic predictor variate, and all misinformation categories significantly correlated with the synthetic criterion variate.

**Table 4:** Standardized function and structure coefficients for the first and second canonical variates.

Predictors	Function		Structure	
	CV1	CV2	CV1	CV2
**Individual differences**				
Conspiracy mentality	0.29	−0.18	**−0.40**	**−0.58**
Credibility of science	−0.27	−0.23	**−0.70**	−0.27
Political issues index	**−0.69**	0.25	**−0.93**	−0.18
SDO	**−0.33**	**0.55**	**−0.71**	**0.40**
Traditionalism	−0.13	**−0.83**	**−0.46**	**−0.79**
**Kinds of misinformation**				
Severity/Spread	0.18	**−1.70**	**−0.85**	**−0.37**
Treatment/Prevention	−0.02	−0.21	**−0.89**	−0.20
Conspiracies	**−1.02**	**0.34**	**−1.00**	−0.09
Misc.	−0.13	**1.50**	**−0.87**	0.24

*N* = 296. SDO, social dominance orientation. Bolded function items are substantial contributors to the synthetic variate. Bolded structure items are significantly correlated with the synthetic variate.

For the second function produced by the canonical analysis (see Table 4), the synthetic predictor was substantially composed of participant scores on the measure of SDO and the measure of Traditionalism, with standardized function coefficients of at least |0.55|. The second function’s synthetic criterion variate was primarily composed of intention to spread misinformation regarding the severity and spread of COVID-19, COVID-19 conspiracies, and miscellaneous COVID-19 misinformation claims. Each criterion variable possessed standardized function coefficients of at least |0.34| for the second synthetic criterion variate. The second model produced by the canonical analysis revealed that individuals high in SDO and low in Traditionalism were less inclined to share misinformation claims regarding the severity and spread of COVID-19, but more inclined to share COVID-19 conspiracies and miscellaneous COVID-19 misinformation claims (see Figure 1). Additionally, the standardized structure coefficients revealed that participant scores on the Conspiracy Mentality Questionnaire and Traditionalism scale were significantly negatively correlated with the synthetic predictor variate and scores on SDO measure significantly positively correlated with the synthetic variate, whereas inclination to share misinformation pertaining to COVID-19 severity and spread correlated negatively with the synthetic criterion variate.

## Discussion

The global COVID-19 pandemic has contributed to an environment allowing for the opportunistic study of the diffusion of misinformation over social media. We report on a preregistered exploratory study investigating theoretically relevant individual differences and willingness to spread different kinds of misinformation on a salient scientific topic, COVID-19. Overall, our canonical model revealed two distinct profiles predicting two patterns of willingness to share misinformation.

The first profile showed that individuals who are both more aligned with liberal policy positions and less oriented toward social dominance were substantially less willing to spread conspiracy-themed misinformation on social media. Whereas prior research has found that conservatism is positively related to spreading political misinformation on social media (Guess et al., 2019), our results suggest that liberals with a low disposition toward social dominance are less willing specifically to share conspiratorial misinformation than are conservatives with a high disposition toward social dominance, at least regarding a culturally salient scientific topic. This finding fits with recent research exploring the relationship between political ideologies, conspiracist ideation, and negative-biased credulity. Generally, the more conservative an individual is the more likely they are to endorse conspiracy theories and to hold a stronger general conspiracist worldview than for individuals who are more liberal, at least for political conservatism as practiced in the United States (van der Linden et al., 2020). Additionally, research by Samore et al. (2018) has found that even when political power dynamics favor conservatives, there exists a positive association between conservatism and conspiracist ideation. The results of our canonical analysis add to the growing body of literature that suggests that political conservatism, at least within the United States, may be partially defined by a conspiracist mindset.

The second profile showed that individuals who are both high in SDO and low in traditionalism are less willing to spread misinformation about the severity and spread of COVID-19, but more willing to spread conspiracy-themed misinformation, as well as miscellaneous culturally salient misinformation claims. This result is particularly interesting in light of prior research indicating that traditionalism, more so than covarying social dominance inclinations, drives pathogen sensitivity (Tybur et al., 2016). Here, we found that individuals high in traditionalism and low in social dominance were more willing to share misinformation about the severity and spread of the COVID-19 pathogen, consistent with the hypothesis that traditionalism functionally relates to pathogen-sensitivity. Equally suggestively, a reverse pattern was obtained with regard to SDO and propensities to spread misinformation, such that individuals who favored social dominance but not traditionalism were less inclined to spread claims about the severity of illness, instead showing a willingness to spread conspiratorial claims, a thematically consistent association insofar as conspiracies inherently entail certain groups vying for advantage over others.

The significant structure coefficients for both profiles hint that the relationships between the selected individual difference variables and the subtypes of COVID-19 misinformation studied here are more complicated than could be revealed by the use of a general linear model approach. However, it is important to note that because of the nature of canonical analysis, the resulting models were algorithmically determined to explain the largest amount of variance, irrespective of the variates’ theoretical context. Although every individual difference selected for inclusion in the present study was motivated by relevant prior literature, follow-up research is needed to validate the patterns of individual differences and misinformation-sharing inclinations reported here. In addition, many other variables likely relevant to a person’s willingness to act as a vector for misinformation spread on social media were not included in the present study, such as degree of media literacy (Guess et al., 2019) or cognitive sophistication (Pennycook and Rand, 2020). Future research should expand the scope of individual differences examined. Further, we investigated only self-reported willingness to share, and did not collect any data related to actual sharing behaviors. Although prior research has found a moderate positive correlation between self-reported willingness to share information and actual rates at which that information is shared online (Mosleh et al., 2020), collecting behavioral data on who actually does share what kinds of specific misinformation is needed.

Another potential limitation of this research concerns our categorization scheme for the claims we tested. Our approach to categorizing coronavirus claims was qualitative and largely influenced by a categorization scheme created for the general public to navigate a fact-checking website. Although the scheme we used produced subscales with acceptable reliability coefficients, resulting in orthogonal models from the canonical analysis, other categorization schemes also warrant future investigation. For example, Pennycook et al. (2020) categorized 21 coronavirus misperceptions using the categories “Optimistic,” “Pessimistic,” “Magical,” and “Conspiratorial” for their investigation about motivated reasoning and political polarization regarding coronavirus claims. Future research might examine additional categorization schemes.

## Conclusion

The present study was exploratory by design. Accordingly, these results should be interpreted with caution, but may inform more sophisticated research and modeling into misinformation diffusion about a scientific topic. Despite the limitations of the present research, we find that factors primarily related to individuals’ political beliefs, and in particular tendencies toward social dominance, are important for understanding how misinformation concerning COVID-19 diffuses online.

## Data Availability Statement

The datasets presented in this study can be found in online repositories. The names of the repository/repositories and accession number(s) can be found in the article/supplementary material.

## Ethics Statement

The studies involving human participants were reviewed and approved by University of California, Merced’s Office of Research and Economic Development. Written informed consent for participation was not required for this study in accordance with the national legislation and the institutional requirements.

## Author Contributions

EL and MP designed the study and created the stimuli. EL carried out statistical analysis. EL and CH contributed to interpreting the findings. EL drafted the manuscript. LP and CH contributed to the final version of the manuscript, providing critical feedback. LP supervised the project. All authors contributed to the article and approved the submitted version.

### Conflict of Interest

The authors declare that the research was conducted in the absence of any commercial or financial relationships that could be construed as a potential conflict of interest.
